# Comparison of age-specific hospitalization during pandemic and seasonal influenza periods from 2009 to 2012 in Taiwan: a nationwide population-based study

**DOI:** 10.1186/s12879-016-1438-x

**Published:** 2016-02-24

**Authors:** Shew-Meei Sheu, Ching-Fang Tsai, Hsin-Yi Yang, Hui-Wen Pai, Solomon Chih-Cheng Chen

**Affiliations:** Department of Medical Research, Ditmanson Medical Foundation Chia-Yi Christian Hospital, Chiayi City, Taiwan; Department of Geriatrics, Ditmanson Medical Foundation Chia-Yi Christian Hospital, Chiayi City, Taiwan; Department of Pediatrics, School of Medicine, Taipei Medical University, Taipei, Taiwan

**Keywords:** 2009 H1N1 pandemic, Seasonal periods, Age-specific hospitalization rate

## Abstract

**Background:**

Determining the age-specific hospitalization burden associated with seasonal influenza and the (H1N1) 2009 pandemic is important for the development of effective vaccine strategies and clinical management. The aim of this study was to investigate age-specific differences in hospitalization rates during the pandemic and seasonal periods.

**Methods:**

Using the Taiwan National Health Insurance Research Database (NHIRD), we identified hospitalized patients with a principle discharge diagnosis of influenza-related infection (ICD-9-CM 487) between 2009 and 2012.

**Results:**

Based on the time distribution of influenza-related hospitalizations and previously reported epidemic periods, the first and second waves of the (H1N1) 2009 pandemic (p1 is known as 2009.07-2010.01, and p2 is known as 2010.12-2011.03) and three seasonal periods (s1 is known as 2010.03-2010.11, s2 is known as 2011.10-2012.03, and s3 is known as 2012.04-2012.10) were found. During these five periods, children younger than 7 years of age consistently had the highest hospitalization rate of the studied age groups. In individuals younger than 50 years of age, the seasonal periods were associated with a significantly lower risk of hospitalization than that of p1 (Relative risk (RR) range = 0.18–0.85); however, they had a significantly higher hospitalization risk for adults over 50 years of age (RR = 1.51–3.22). Individuals over 50 years of age also had a higher intensive care unit admission rate and case fatality ratio than individuals under than 50 years of age during the seasonal periods and especially during the pandemic periods.

**Conclusions:**

In both pandemic and seasonal periods, the highest hospitalization rate was observed for children younger than 7 years of age. Adults over 50 years of age had a higher hospitalization risk during the seasonal periods and a higher clinical severity during the pandemic periods. Those results emphasize that the importance of influenza-related prevention strategies in the younger and older age groups, either seasonal or pandemic periods.

## Background

Influenza infection causes severe morbidity and mortality around the world [1]. Although the influenza-associated disease burden and use of medical facilities can be reduced by effective vaccination strategies [2], the influenza virus is characterized by antigenic alterations that may lower vaccine efficacy [3]. Pandemic influenza arising from novel subtypes of the virus further increases disease severity [4]. Therefore, the epidemiological characteristics of pandemic and seasonal influenza periods can aid in the development of prevention strategies and clinical management.

Seasonal influenza A (H3N2 and H1N1) and influenza B have been the three major circulating virus subtypes in humans for many years [1]. In 2009, a new strain of influenza A, namely, pandemic A (H1N1) 2009 virus (A(H1N1)pdm09), spread rapidly to different countries. In 2010–2011, a second wave of the pandemic H1N1 infection occurred in Hong Kong, the UK, Wisconsin and Taiwan [5–8]. From 2009 to 2012 in Taiwan, there were five epidemic periods. The dominant individual circulating virus was A(H1N1)pdm09 from July 2009 to January 2010, influenza B and subsequent A(H3N2) from March 2010 to November 2010, A(H1N1)pdm09 from December 2010 to March 2011, influenza B from October 2011 to March 2012 and A(H3N2) from April 2012 to October 2012 [7–9].

Hospitalized patients from the (H1N1) 2009 pandemic period were younger than patients from the seasonal periods [10–13]. Moreover, during the (H1N1) 2009 pandemic, a high proportion of deaths occurred in individuals under 60 years of age, whereas during the seasonal periods, a high proportion of deaths occurred in individuals 65 years and older [14–16]. Not all studies included data on younger children or information about age distributions in their analyses of influenza-related hospitalizations [10, 11]. Comparisons of the hospitalization rate and the severity of infection during the 2009 pandemic and seasonal periods have been presented in several studies [10–17]. However, few studies have compared the age-specific hospitalization burden associated with seasonal influenza and pandemic periods [12, 13].

Nationwide population-based data can provide valuable information on the disease burden and the impact of influenza infection on health. Using the Taiwan National Health Insurance Research Database (NHIRD), we aimed to evaluate the age-specific hospitalization rates and clinical severity associated with influenza during the pandemic and during seasonal periods between 2009 and 2012.

## Methods

### Data source

Population-based data consisting of pneumonia and influenza cases from 2002 to 2012 were obtained from the Taiwan NHIRD. The NHIRD is a claims database that collects nationwide outpatient/emergency and inpatient data and has been used extensively in many studies [18]. The National Health Insurance (NHI) program, which was implemented on March 1, 1995, provides universal health care for all legal residents and covers over 99 % (approximately 23 million) of the population in Taiwan. By using encrypted identification numbers, we retrieved admission records and linked all information on patient characteristics and prescriptions from 2009 to 2012. In each admission record, up to five discharge diagnoses were available and coded using the International Classification of Diseases, Ninth Revision, Clinical Modification (ICD-9-CM). This study was reviewed and approved by the Institutional Review Board of the Ditmanson Medical Foundation of Chia-Yi Christian Hospital, Taiwan.

### Study subjects

In cases of hospitalization, the discharge code ICD-9-CM 487 was more specific for influenza-related infection than the 480–487 codes (which include pneumonia and influenza). ICD-9-CM code 487 was used in the principle diagnosis at discharge, and it was used to identify episodes of influenza-related hospitalization. Patients who had been hospitalized because of influenza infection between 2009 and 2012 were included. During the study period, five epidemic periods were defined according to the influenza-positive rate from the laboratory confirmation of the Taiwan Centers for Disease Control (CDC) surveillance network [7–9]. The epidemic periods occurred from July 2009 to January 2010 (pandemic period, p1), March 2010 to November 2010 (seasonal period 1, s1), December 2010 to March 2011 (the second wave of pandemic, p2), October 2011 to March 2012 (seasonal period 2, s2) and April 2012 to October 2012 (seasonal period 3, s3). To compare the difference in the rates of age-specific hospitalization and clinical severity between the pandemic and seasonal periods, we stratified the patients by age for each of the five study periods. Because Taiwan has implemented a Nationwide In-school Influenza Vaccination strategy, school-aged children have a much higher vaccination rate than children under the age of 7. Therefore, the patients were categorized into five age groups (0–6, 7–12, 13–18, 19–49, 50–64 and ≥ 65 years of age) to investigate the effect of the Nationwide In-school Influenza Vaccination strategy.

### Statistical analysis

We calculated the age-specific hospitalization rate among the five epidemics between 2009 and 2012. The numerator of the hospitalization rate was the number of incident cases, and the denominator was the population of a specific age group during that year, which was obtained from the Department of Statistics in the Ministry of the Interior for Executive Yuan in Taiwan. The hospitalization rate of specific age groups during p1 was used as the reference, which allowed for a successful comparison of the hospitalization rate between two pandemic periods. Using p1 as the reference also allowed an investigation of the change between the seasonal and pandemic periods by using the same reference. The relative risk (RR) and 95 % confidence interval (CI) were used to estimate the hospitalization rates between periods using Poisson regression. The intensive care unit (ICU) admission rate and the case fatality ratio (CFR) represented the clinical severity of hospitalized influenza cases during different periods. The age-specific CFR was the percentage of fatal cases among the number of hospitalized influenza cases in a particular age group. Data analysis was performed with SPSS software (version 21.0; IBM Corporation, Somers, NY, USA). Two-tailed P-values that were less than 0.05 were considered to be statistically significant.

## Results

**Fig. 1:**
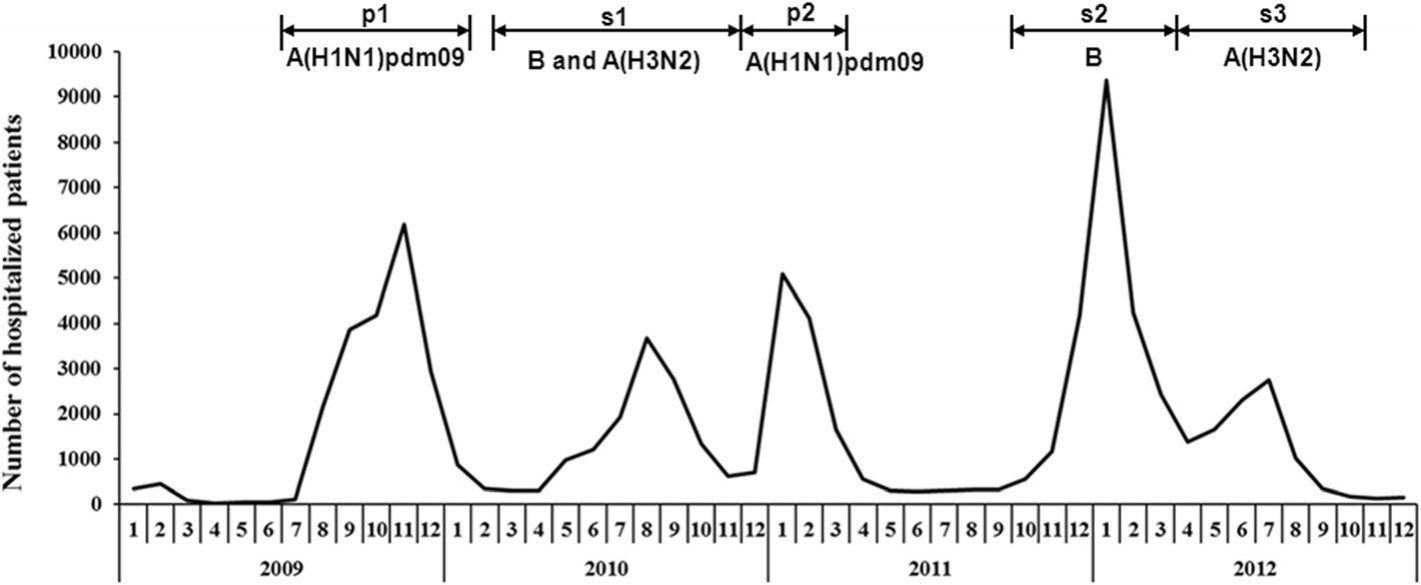
Number of influenza-related hospitalizations during the study period between 2009 and 2012 in Taiwan. Five epidemic periods were defined according to the laboratory-confirmed influenza-positive rate from the Taiwan CDC surveillance network, and the dominant isolated viruses were A(H1N1)pdm09 (p1), influenza B and subsequent A(H3N2) (s1), A(H1N1)pdm09 (p2), influenza B (s2) and A(H3N2) (s3) [7–9]

According to the time distribution of influenza-related hospitalization, five epidemics were defined during the study period between 2009 and 2012 (Fig. 1). Among the periods, the numbers of hospitalized cases were 19,470 (p1), 12,576 (s1), 10,013 (p2), 19,646 (s2), and 9,482 (s3). The s2 seasonal period was the period with the largest epidemic.

### Age-specific hospitalization rates among the five periods

**Fig. 2:**
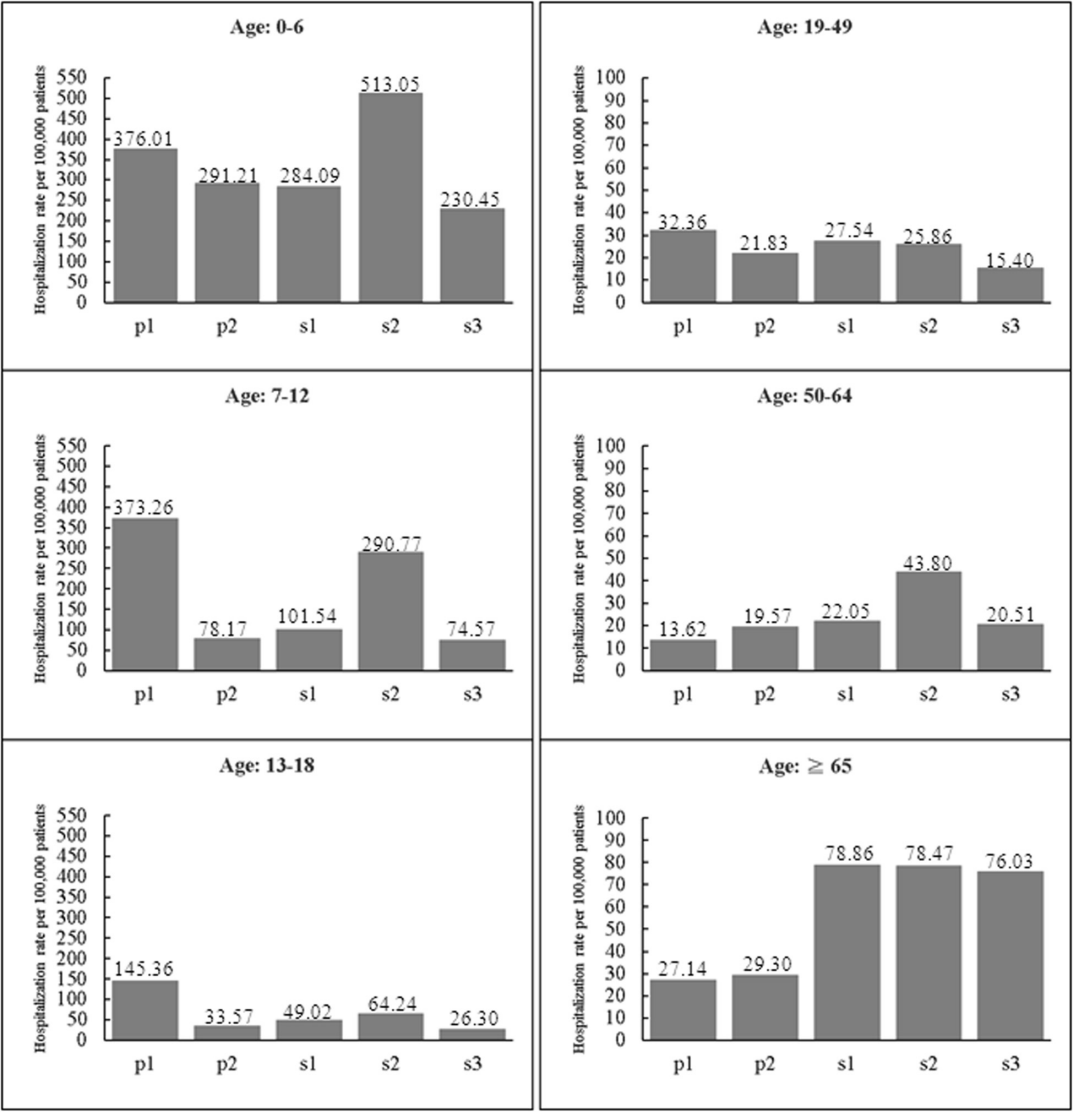
Age-specific hospitalization rate during the five periods. The periods p1 and p2 were pandemic periods, whereas s1, s2 and s3 were seasonal periods

To investigate the age-specific hospitalization burden among the five periods, the hospitalization rates were further stratified by age. Compared with other age groups, the group of children younger than 7 years of age had the highest hospitalization rate in both the pandemic and seasonal periods; the hospitalization rate during s2 was markedly higher for patients younger than 7 years of age and between 50 and 64 years of age (Fig. 2). The hospitalization rate during p1 was the highest among the 7-12-, 13-18- and 19-49-year-old age groups. Moreover, for the over-50 age groups, the hospitalization rates during the seasonal periods (s1, s2 and s3) were higher than they were during the pandemics.

### RR of age-specific hospitalization

**Fig. 3:**
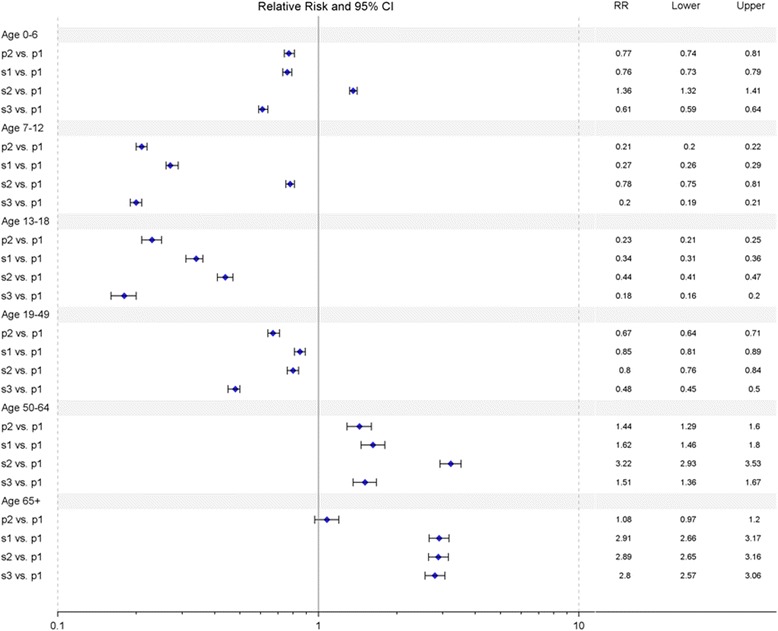
Relative risk (RR) comparison between p1 and the remaining four epidemic periods. Age-specific RRs and 95 % CIs were estimated by Poisson regression

To compare the RR of age-specific hospitalization between different periods, we used the age-specific hospitalization rates during p1 as the reference (Fig. 3). In the under-7 age group, the risk of influenza-related hospitalization was significantly lower during p2, s1 and s3 (RR = 0.77, 0.76 and 0.61, respectively), whereas during s2, the under-7 age group had a higher risk of hospitalization (RR = 1.36, 95 % CI = 1.32-1.41). Among the 7-12-, 13-18- and 19-49-year-old groups, periods p2, s1, s2 and s3 were associated with a lower risk of hospitalization (RR range = 0.18-0.85), whereas there was much lower risk during p2, s1 and s3 in the 7-12- and 13-18-year-old groups. In the over-50 age groups, the hospitalization risk during the three seasonal periods and p2 was consistently higher than it was during p1 (Fig. 3), except in patients over 65 years of age during p2 (RR = 1.08, 95 % CI 0.97-1.20).

### Comparison of age-specific clinical severity among the five periods

The clinical severity observed during the five periods was assessed on the basis of the ICU admission rate and CFR. The ICU admission and CFR were stratified by age, and then a higher ICU admission rate and an age-specific CFR occurred in the older age groups (50–64 and 65 years and older) (Table 1). Among the three groups of people over 19 years of age, the ICU admission rate and age-specific CFR during the pandemic periods were higher than they were during the seasonal periods, and they were higher in p2 than in p1 (Table 1).

**Table 1:** The ICU admission rate and CFR categorized by age in the five epidemics from July 2009 to October 2012

	≤6		7-12		13-18		19-49		50-64		≥65	
Epidemics	ICU %	CFR	ICU %	CFR	ICU %	CFR	ICU %	CFR	ICU %	CFR	ICU %	CFR
p1	1.49	0.04	0.8	0.03	1.21	0.00	2.73	0.43	9.88	1.23	10.34	4.50
p2	1.7	0.10	1.85	0.16	1.69	0.15	4.87	0.56	13.77	3.73	13.17	5.90
s1	1.26	0.03	0.5	0.00	0.84	0.00	1.53	0.22	4.76	1.04	6.57	2.55
s2	0.74	0.06	0.4	0.09	1.4	0.17	1.84	0.17	4.33	1.25	9.78	3.13
s3	1.3	0.06	0.64	0.18	0	0.00	2.19	0.29	3.18	0.31	8.85	2.63

ICU: intensive care unit

CFR: case fatality ratio

Periods p1 and p2 were pandemic periods

Periods s1, s2 and s3 were seasonal periods

The ICU admission rate was calculated by taking the number of ICU cases divided by the number of hospitalized cases

The CFR was calculated by taking the number of fatal cases divided by the number of hospitalized cases

## Discussion

This nationwide population-based study compared the age-specific hospitalization rates between seasonal influenza periods from 2009 to 2012 and the hospitalization rate during the (H1N1) 2009 pandemic in Taiwan. Among the five periods, the largest number of patients was admitted during seasonal period s2; the younger than 7 years of age group had the highest hospitalization rate during both the pandemic and seasonal periods. For p1, individuals younger than 50 years of age had a higher rate of influenza-related hospitalization when compared with other periods. Compared with the age-specific hospitalization rate during p1, the 50–64-year-old group had a significantly higher risk of hospitalization during p2 as well as a higher ICU admission rate and CFR. Moreover, there was a significantly higher risk of hospitalization during the seasonal periods for individuals over 50 years of age. By contrast, this age group had a higher ICU admission rate and CFR during the pandemics compared with the seasonal periods.

In our study, s2 had the largest epidemic between 2009 and 2012 and led to more hospitalized patients than the pandemics (Fig. 1). This time period corresponded to the influenza season that was dominated by influenza B in 2011–2012 [9]. During this time, Yam88-lineage influenza B was the only one to emerge in Taiwan, whereas Vic87-lineage influenza B and influenza A (H3N2) were dominant in other regions of the world [9, 19]. The timing and intensity of the influenza B epidemic may be related to the size of the susceptible population combined with the incidence of vaccine mismatch. The (H1N1) 2009 pandemic limited the activity of influenza B such that its prevalence was low in 2009 and 2010. This limited activity likely led to an increase in the size of the population that was susceptible to influenza B [9]. Furthermore, between 2009 and 2012, the influenza vaccine that only contained the Victoria lineage of influenza B was recommended by the World Health Organization for use in Taiwan [9]. This finding shows that effective vaccine strategies and the continued observation of virus activity are important for minimizing the impact of seasonal influenza.

Pandemic period p1 was associated with a high hospitalization rate for individuals younger than 50 years of age (Fig. 2), which is consistent with the results in Hong Kong [13], Australia and the USA [12]. Additionally, when compared with the rate of age-specific hospitalization during p1, a significantly higher risk of hospitalization during p2 occurred for individuals from 50 to 64 years of age, whereas a much lower risk of hospitalization during p2 was observed in the 7-12- and 13-18-year-old age groups (Fig. 3). Among those age groups, children may have been protected during p2 by the immunity they acquired during p1 because children made up the major hospitalization groups during that period (Fig. 2). Moreover, the one-dose coverage rate of the A(H1N1)pdm09 monovalent vaccine reached 72 % in children aged 7–18 years from November 2009 to March 2010, which provided further immune protection in this age group during p2 [7, 8]. Additionally, the government-funded vaccination program was not expanded to individuals aged 19–64 years; the older subset of this age group (50–64 years) may have had a higher frequency of influenza-related hospitalization because of their higher rate of underlying medical conditions compared with younger individuals [20]. These factors could explain the difference in the rate of age-specific hospitalization between p1 and p2.

In comparison with the pandemic period, the seasonal periods displayed a higher hospitalization rate and risk for individuals aged 50 years and older (Figs. 2 and 3). For the (H1N1) 2009 pandemic, the relatively lower risk of hospitalization among individuals born prior to 1957 (~52-91 years of age in 2009) has been explained by their preexisting immunity because of their first exposure to influenza A (H1) during childhood [14, 21]. According to a study in Hong Kong, a higher hospitalization rate for seasonal influenza occurred in adults aged 65 years and older when compared with that rate for the (H1N1) 2009 pandemic [13]. Our results further demonstrated that middle-aged adults (50–64 years) in Taiwan also experienced higher hospitalization rates during the seasonal periods than during the (H1N1) 2009 pandemic. Among the three seasonal periods in our study, the rates of influenza-related hospitalizations in adults aged 50–64 years ranged from 21 to 44 per 100,000 patients, which are similar to values reported in Australia (33.3 per 100,000) [22] and the United States (37.9 per 100,000) [23]. A comparison of influenza-related hospitalization between countries showed that the hospitalization rates of adults aged 50–64 years were similar but hospitalization rates in adults aged 65 and older varied between countries, with rates of 157.4, 205 and 63 per 100,000 reported in Australia [22], the United States [23] and Switzerland [24], respectively. The reason for this discrepancy is not clear. Many factors may contribute to the difference, such as population demographics, vaccination coverage, and the time period considered [22].

The ICU admission rates during the pandemic in different countries were variable (9.1–31.0 %) and were always calculated for the overall population [11, 20, 25]. A study based on the 51 Canadian Nosocomial Infection Surveillance Program hospitals revealed that the ICU admission rate was significantly higher during the pandemic period [11]. Although a previous study demonstrated that age is significantly associated with a higher risk of ICU admission [25], our result demonstrated that the ICU admission rate for individuals over 19 years of age was higher during the pandemic than during the seasonal periods (Table 1). Additionally, the age-specific CFR in individuals over 19 years of age was notably higher in p2 than in p1 (Table 1), which is comparable with the increased severity of the second wave of the (H1N1) 2009 pandemic in Hong Kong and Wales, UK [5, 26]. The higher age-specific CFR in individuals over 50 years of age occurred during the pandemics (Table 1), although this finding was different from the high proportion of deaths in individuals under 60 years of age that occurred during the (H1N1) 2009 pandemic [14, 15]. Reports from Germany and Austria also showed that the highest CFR during the pandemic occurred in individuals aged 65 years and older [25, 27], which is similar to what happened during the seasonal periods [28]. Therefore, the fatalities among different age groups and in different countries were inconsistent during the 2009 pandemic periods.

The retrieval of data from the Taiwan NHIRD was a limitation of the present study because of a lack of confirmatory laboratory testing to identify cases. However, we used the specific ICD-9-CM code 487 that was denoted in the principle discharge diagnosis codes to identify episodes of influenza-related hospitalization. The epidemic periods of influenza-related hospitalization from 2009 to 2012 were comparable with data based on the laboratory confirmation of the Taiwan CDC surveillance network [7–9]. The inclusion of additional influenza seasons would be helpful. Unfortunately, only pneumonia and influenza records for 2009–2012 were available in the dataset purchased by our hospital from NHIRD. We hope to expand this analysis using data from other influenza seasons in the future.

## Conclusions

During both the pandemic and seasonal periods, the highest hospitalization rate was observed in children younger than 7 years of age. Adults over 50 years of age had a higher hospitalization risk during the seasonal periods and a higher clinical severity during the pandemic periods. These results suggest the importance of implementing influenza vaccination programs for children younger than 7 years of age and for individuals over 50 years of age during both seasonal and pandemic periods.

## Abbreviations

CDC: centers for disease control
CFR: case fatality ratio
ICD-9-CM: International Classification of Diseases, Ninth Revision, Clinical Modification
ICU: intensive care unit
NHIRD: Taiwan National Health Insurance Research Database
